# Cytotoxic Activity and Lymphocyte Subtypes in Mice Selected for Maximal and Minimal Inflammatory Response after Transplantation of B16F10 and S91 Melanoma Cells

**DOI:** 10.1155/2022/3298542

**Published:** 2022-02-28

**Authors:** Lindsey Castoldi, Graziela Gorete Romagnoli, Marjorie de Assis Golim, Orlando Garcia Ribeiro, Olga Célia Martinez Ibañez, Durvanei Augusto Maria, Andréa Vanessa Pinto Domeneghini, Maria Carolina Gameiro, Priscila Raquel Martins, Martha Maria Mischan, Ramon Kaneno

**Affiliations:** ^1^Health Sciences Institute, Federal University of Mato Grosso-UFMT, Sinop, Mato Grosso, Brazil; ^2^Department of Chemical and Biological Sciences, Institute of Bioscience of Botucatu, São Paulo State University-UNESP, Botucatu, São Paulo, Brazil; ^3^Department Health Science, Oeste Paulista University-UNOESTE, Jaú, São Paulo, Brazil; ^4^Hemocentro Division, School of Medicine of Botucatu, São Paulo State University-UNESP, Botucatu, São Paulo, Brazil; ^5^Laboratory of Immunogenetics, Institute Butantan, São Paulo, São Paulo, Brazil; ^6^Laboratory of Biochemistry and Biophysics, Institute Butantan, São Paulo, São Paulo, Brazil; ^7^Central Paulista University Center - UNICEP, São Carlos, São Paulo, Brazil; ^8^Department of Biostatistics, Institute of Bioscience of Botucatu, São Paulo State University-UNESP, Botucatu, São Paulo, Brazil

## Abstract

AIRmax and AIRmin mice strains were selected according to the intensity of their acute inflammatory responsiveness. Previous studies have shown that AIR mice differ in their resistance to chemically induced skin tumors and in the development of melanoma metastases, in addition to differences in neutrophil and NK cells activity. In the present work, we aimed to evaluate whether the difference of susceptibility to murine melanoma is associated with NK cytotoxic activity against Yac.1 cells and lymphocyte subsets. Mice were subcutaneously inoculated with B16F10 or S91 melanoma cells. After 7, 14, or 30 days, the animals were euthanized to analyze the number of lymphocyte subsets, cytotoxic activity, and number of cytokine-producing spleen cells. AIRmax mice presented a higher number of CD4^+^/CD25^+^ cells than that of AIRmin mice following inoculation of B16F10 cells, whereas inoculation of S91 cells reduced CD4^+^/CD25^+^ and increased TCD8^+^ cell subsets in the AIRmax mice. AIRmax mice had a higher number of interleukin (IL)-10- and IL-12-producing cells and a lower number of interferon-*γ*–producing cells than those of AIRmin mice at 30 days. The cytotoxic activity of nonadherent spleen cells was similar in both the AIR strains. These results suggest that melanoma cells can induce different responses in AIR mice, possibly owing to alterations in regulatory mechanisms, such as the action of CD4^+^/CD25^+^ regulatory T cells and IL-10, in AIRmax mice.

## 1. Introduction

AIRmax and AIRmin mice were obtained by bidirectional genetic selection for high (max) or low (min) acute inflammatory response (AIR) [1]. Selective breeding was performed from a highly polymorphic population (F0) obtained by intercrossing eight inbred mouse strains (A, DBA2, P, SWR, SJL, CBA, BALB/c, and C57BL/6) [1]. This selection was based on plasma protein exudation and local leukocyte influx following the subcutaneous injection of polyacrylamide beads [1]. The progressive divergence between AIRmax and AIRmin mouse lines following successive generations of selective breeding achieved a 30-fold difference in leukocyte infiltration and a 2.5-fold difference in exudate protein concentrations [2]. These differences reflect the accumulation of alleles with opposite and additive roles in the inflammatory response, making this murine model useful for studying the genetic control of the inflammatory process and nonspecific immunity in the development of infectious, autoimmune, and neoplastic diseases [2–6].

Genetic studies indicated that the contrasting inflammatory responsiveness of AIR strains involves at least 11 QTLs (quantitative trait loci) and specific genes/alleles [7]. The solute carrier 11a1 gene (*Slc11a1*) on chromosome 1 is responsible for the transport of ions (iron, zinc, and manganese) in phagocytic cells, interfering with macrophage activation, and it significantly modulates the differential response between AIR mice strains to intracellular pathogens, tumorigenesis, and wound healing [8, 9]. Genes involved in DNA repair mechanisms may have been differently selected in AIRmax and AIRmin mice, like polymorphism of *Ahr* (aryl hydrocarbon receptor) alleles found in these lines which confer low or high affinity to polycyclic aromatic hydrocarbons and, respectively, resistance or susceptibility to chemical carcinogenesis [10]. Vorraro et al. [11] and Galvan et al. [12] mapped a major locus named *Irm1* (major inflammatory response modulator 1) on chromosome 7, and *Irm2* on chromosome 5, linked to the number of infiltrating cells through the production of interleukin (IL)-1 beta.

So, in that context, AIRmax mice produce higher levels of growth and chemotactic factors for neutrophils than those of AIRmin mice [13]. Furthermore, AIRmax neutrophils were more resistant to apoptosis [13]. AIRmax is more resistant to infection by *Salmonella enterica* serotype Typhimurium [4], *Trypanosoma cruzi* [14], and *Paracoccidioides brasiliensis* [15]. Contrarily, AIRmax is susceptible to pristane-induced arthritis [5], experimental autoimmune uveitis [16], and IgA glomerulonephritis experimental model [17].

As mentioned, selective breeding for acute inflammatory reactivity may have provided the selection of tumorigenesis-related factors [6, 10, 18–21]. Biozzi et al. [18] observed that AIRmax mice are more resistant than AIRmin mice to the development of chemical skin carcinogenesis induced by 9,10-dimethyl-1,2-benzantracene (DMBA) and 12-O-tetradecanoyl-phorbol-13-acetate and exhibit a lower incidence and tumor multiplicity. AIRmax mice showed higher resistance than AIRmin mice to the development of urethane-induced lung tumorigenesis [19], as well as to adenoma and adenocarcinoma induced by DMBA [20] and 1,2-dimethylhydrazine (DMH) [21]. Furthermore, AIRmax mice are resistant to the development of metastases in murine (B16F10) and human (SKMel-28) melanoma, while treatment with nonsteroidal anti-inflammatory drugs (aspirin and nimesulide) alters this profile, increasing the incidence of metastasis by 60% [6]. Despite this, cancer susceptibility in these animals appears to depend on the target organ once AIRmax mice are more susceptible than AIRmin mice to DMH-induced chemical colon carcinogenesis [21].

Previously, we observed that normal AIRmax mice exhibited a larger number of natural killer (NK) (CD49b^+^) cells in the spleen than AIRmin mice, which is associated with a higher cytotoxic activity against Yac.1 cells [22]. Furthermore, they have more TCD8^+^ lymphocytes in the spleen and produce more proinflammatory cytokines, such as interferon-*γ* (IFN-*γ*) and tumor necrosis factor-*α* (TNF-*α*) by spleen cells, than those of AIRmin mice [22].

Knowing that AIRmax and AIRmin mice differ in their resistance to chemically induced skin tumors [18] and to the development of melanoma metastases [6], added to the differences already observed in relation to neutrophils [13] and NK cells [22], in the present study, we analyzed the NK cytotoxic activity against Yac.1 cells and quantified the lymphocyte subsets in AIR mice bearing subcutaneous B16F10 or S91 melanoma cells. We observed that the maximal inflammatory response observed in AIRmax mice is accompanied by regulatory mechanisms, which may influence the immune response to tumor growth.

## 2. Material and Methods

### 2.1. Experimental Design

Groups of 5–8 AIRmax and AIRmin mice were subcutaneously injected with 5 × 10^4^ B16F10 or S91 melanoma cells in the right flank. Normal control animals were injected with saline solution only. On the 7^th^, 14^th^, and 30^th^ day following melanoma cell inoculation, these animals were euthanized for evaluation of immunological parameters. The primary tumor growth was evaluated, and the lung, liver, and kidney were macroscopically analyzed for the occurrence of melanoma.

### 2.2. Animals

Male AIRmax and AIRmin mice, 8–12 weeks old, from generations 43 to 45 of selective breeding were obtained from the Laboratory of Immunogenetics of the Institute Butantan, São Paulo (Brazil). Following acclimatization in the Animal House of the Department of Pathology, School of Medicine of Botucatu, São Paulo State University (Brazil), the animals were randomly distributed into polypropylene cages, with commercial feed and water *ad libitum*, and 12 h dark/light cycles, and kept at a temperature of 22 ± 1 °C. The animals were anesthetized with 4% sodium pentobarbital, and their spleens were removed. All procedures involving animals were performed in accordance with the international guidelines for the care and use of laboratory animals and were approved by the Ethical Committee for Animal Experimentation at the School of Medicine of Botucatu (protocol number 440).

### 2.3. Melanoma Cell Lines

Murine B16F10 melanoma cells were obtained from the Rio de Janeiro Cell Bank (Brazil), and the S91 melanoma cells were provided by the Laboratory of Biochemistry and Biophysics, Institute Butantan. Cells were cultured in DMEM (Cultilab, São Paulo, Brazil) with 25 mM HEPES (Sigma-Aldrich, MO, USA) and 10% heat-inactivated fetal bovine serum (FBS; Cultilab) at 37°C and 5% CO_2_ tension until the monolayer cell was completed, when detached from bottles with 0.5% trypsin (Nutricell, São Paulo, Brazil). After trypsin inactivation with 10% FBS, viable cells were counted, and the cell suspension was adjusted to 5 × 10^5^ cells/mL. Animals were subcutaneously injected with 0.1 mL of the B16F10 or S91 cell suspension. Both tumor lines were used due to MHC compatibility with AIR mice.

### 2.4. Yac.1 Target Cell

Yac.1 cells were cultured in complete culture medium prepared with RPMI 1690 medium (Cultilab) supplemented with 10% FBS, 200 mM L-glutamine, 2% gentamicin, and 25 nM HEPES (Sigma-Aldrich) at 37°C and 5% CO_2_. Cells were washed and suspended to 10^6^ cells/mL in RPMI 1% FBS and used as targets for NK cells in the colorimetric assay for cytotoxic activity analysis.

### 2.5. Colorimetric Assay for Cytotoxic Activity Analysis

The cytotoxic activity of nonadherent spleen cells was evaluated using the nonradioactive colorimetric method based on the measurement of lactate dehydrogenase (LDH) activity (Cytotoxicity Detection Kit, Roche Diagnostics, Basel, Switzerland). Mononuclear cells were obtained by centrifugation of the spleen cell suspension on a Ficoll–Hypaque gradient (Sigma-Aldrich), followed by incubation on glass Petri dishes for 90 min at 37°C to remove the adherent cells. Nonadherent cells were recovered from the Petri dishes, suspended in complete culture medium, and adjusted to 10^7^ cells/mL. For the assay, we placed 50 *μ*L of nonadherent cell suspension (effectors) into a 96-well U-bottom microtiter plate and cultured with 50 *μ*L of the target cell suspension (Yac.1) at a concentration of 10^6^ cells/mL. Maximal lysis of the target cells was determined by adding 100 mL of Triton X (Sigma-Aldrich). Spontaneous lysis of Yac.1 cells was determined by incubation with RPMI+1% FBS. We used RPMI 1% FBS (without cells) as a background control. After 4 h of incubation at 37°C and 5% CO_2_ tension, the plate was centrifuged for 10 min at 1500 rpm, and 50 *μ*L of the supernatant was carefully removed from each well and transferred into a 96-well flat-bottomed microtiter plate (Nunc A/S, Roskilde, Denmark). We quantified LDH activity by adding 50 *μ*L of a diaphorase/NAD^+^ mixture and a dye solution containing iodotetrazolium chloride and sodium lactate into each well. Plates were incubated for 30 min in a dark box at room temperature, and the reaction was read by spectrophotometry at 492 nm (Thermo Electron Corporation, MA, USA). The optical density of samples and controls was used to calculate the percentage of lysis using the following formula: cytotoxicity (%) = ([{target and effector cell mixture − control of effector cells} − spontaneous lysis control]/{control of maximum lysis − spontaneous lysis control}) × 100.

### 2.6. Analysis of Spleen Lymphocyte Subsets

Spleen cell suspensions were obtained by teasing organ fragments on a fine nylon screen. Cells were suspended in complete RPMI medium, washed twice with 0.5% bovine serum albumin isoton, and adjusted to 10^7^ cells/mL. Cells were then distributed into a 96-well U-bottom microtiter plate (100 *μ*L/well) and incubated with 10 *μ*L of normal mouse serum for 10 min. After washing, the cells were incubated with fluorochrome-labeled monoclonal antibodies (mAb) for 60 min. PE-conjugated rat IgM anti-mouse CD49b/Pan-NK, PerCp-conjugated hamster IgG anti-mouse CD3*ε*-chain, FITC-conjugated rat IgG anti-mouse CD4 (L3T4), PE-conjugated rat IgG anti-mouse CD8a (Ly-2), PE-conjugated rat IgG anti-mouse CD25 (IL-2 *α*-chain receptor), and respective isotopic controls were supplied by BD Pharmingen (CA, USA). Data were acquired using a FACSCalibur (Becton Dickinson Immunocytometry Systems, NJ, USA) at the Laboratory of Flow Cytometry of the Blood Bank, School of Medicine of Botucatu, and analyzed using the Cell Quest software. Fluorescence overlap was electronically calibrated using single-color-stained standard beads (FITC, PE, or PerCp), and 10,000 events were acquired and stored for each analysis. Lymphocyte populations were expressed as percentages (10,000 events) of CD3^+^, CD4^+^, CD8^+^, CD4^+^/CD25^+^, CD49b^+^ NK [23], and CD3^+^/CD49b^+^ (nonclassical natural killer T cell (NKT)) [24] cells within the selected gate.

### 2.7. Quantification of Cytokine-Producing Cells by ELISpot

The number of IL-2, IL-6, IL-10, IL-12, TNF-*α*, and IFN*γ*-secreting cells was determined by ELISpot. Briefly, 96-well Immulon microtitration plates (Millipore Merck, Darmstadt, Germany) were sensitized overnight at 4°C with an appropriate capture mAb. After washing, the plates were quenched for 2 h at room temperature with RPMI+10% FBS. After quenching, 2 × 10^6^ spleen cells were added to each well, followed by incubation for 20 h at 37°C under 5% CO_2_ tension. The plates were washed twice with deionized water and three times with phosphate-buffered saline (PBS) + 10% FBS. Then, specific biotinylated mAbs were applied and incubated for 2 h at 37°C. After washing, streptavidin-peroxidase was added to the reaction for 1 h at room temperature. Plates were washed four times with PBS-Tween 20 (0.05%) and twice with fresh PBS. The colorigenic reaction was developed by adding 3-amino-9-etilcarbazole substrate (AEC Substrate Reagent Set for ELISPOT, BD Biosciences, NJ, USA). Plates were washed with fresh tap water, dried at room temperature, and read using an ImmunoSpot Analyzer (BioSys GmbH). Data were expressed as the number of spots/2 × 10^6^ cells.

### 2.8. Statistical Analysis

Microscopic analyses were performed using Fisher's exact test. Cytotoxic activity and percentage of lymphocyte subsets were compared by 3 × 3 factorial analysis of fully randomized data (SAS software system v8 for Windows) followed by Tukey's post hoc test. Data that failed the Levene variance homogeneity test were transformed into log or square root according to the type of relation observed between the mean values and the standard deviation of the groups. In cases in which the transformation was inefficient in stabilizing the variances, we performed a Kruskal–Wallis nonparametric test. Data of cytokine-producing cells were analyzed using the Kruskal–Wallis nonparametric test, followed by Dunn's test for multiple comparisons. All data were considered significant at *P* < 0.05.

## 3. Results

### 3.1. Analysis of Tumoral and Metastasis Development

Tumor masses >1 mm were considered for the tumoral growth analysis. The B16F10 (H-2^b^) and S91 (H-2^d^) melanoma were selected for these experiments because of their H-2 haplotypes. AIRmax mice are mainly H-2^b^, whereas AIRmin mice are H-2^d^ or H-2^k^ [6]. Although there was no statistical difference, macroscopic analysis of the primary tumor incidence revealed that AIRmax mice were more susceptible than AIRmin mice to the development of both B16F10 and S91 (Table 1). We found no macroscopic melanomas in the thoracic or abdominal organs of mice inoculated with B16F10 or S91 cells.

### 3.2. Cytotoxic Activity

**Figure 1:**
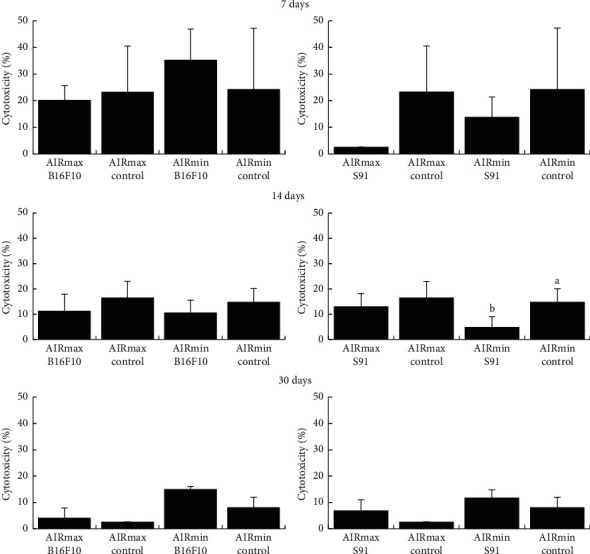
Cytotoxic activity of effector nonadherent spleen cells of acute inflammatory response (AIR) mice (*n* = 6) using lactate dehydrogenase activity released into the supernatant from the cytosol of damaged cells. The results are expressed as a mean percentage and standard deviation of specific lysis against the Yac.1 target cells (effector to target cell ratio 10:1). Lowercase letters indicate comparison of cytotoxic activity within the same AIR mouse line (*a* > *b*, *P* < 0.05); capital letters indicate a comparison of cytotoxic activity between AIRmax and AIRmin (*A* > *B*, *P* < 0.05).

Cytotoxic activity was measured using Yac.1 cells as the target cell for the LDH colorimetric assay. Yac.1 cell is a mouse T-cell lymphoma cell line that is induced by inoculation of the Moloney leukemia virus into a newborn A/Sn mouse and is highly sensitive to lysis by NK cells and is widely used as a target cell for the determination of cytotoxic activity of mice NK cells [25, 26]. In addition, the spleen is the largest lymphoid organ with the highest lymphocyte throughput of all lymphatic tissues, and it is the site of cell pooling, elimination of unnecessary cells, and regulatory effects on a wide variety of immune system cells, including NK cells (CD49b^+^) [23, 27]. Thus, it is possible to consider that the cytotoxic activity evaluated in this work is related to NK cells from AIR mice, since there was no previous stimulation of animals with Yac.1 cells in the mice.  Figure 1 shows that melanomas do not induce significant alterations in NK activity between AIRmax and AIRmin mice during tumor development. At 14 days, the AIRmin control showed higher NK activity than the AIRmin S91 melanoma (14 days: AIRmin S91: 4.94 ± 4.09% vs control: 14.85 ± 5.25%).

### 3.3. Analysis of Lymphocyte Subsets

Lymphocyte subtypes were analyzed to verify whether tumor development favors a specific cell population, which could explain the differential susceptibility to tumorigenesis observed in these animals. As presented in Table 2, AIRmax and AIRmin mice show similar counts of CD3^+^, CD4^+^, CD49b^+^, and CD3^+^/CD49b^+^ lymphocyte subtypes at all time points evaluated independently of the cell line injected. We observed that B16F10 induced a higher percentage of CD4^+^/CD25^+^ in AIRmax mice than in AIRmin mice at 7 and 14 days. Conversely, S91 cells induced higher CD4^+^/CD25^+^ cell levels in AIRmin mice than in AIRmax mice after 7 days of tumor development. CD8^+^ cells were higher in AIRmax mice than in AIRmin mice at 14 days of S91 melanoma growth. In the control groups, AIRmax mice showed a higher number of CD3^+^ and CD8^+^ cells than that of AIRmin mice.

### 3.4. Cytokine-Producing Cells

**Figure 2:**
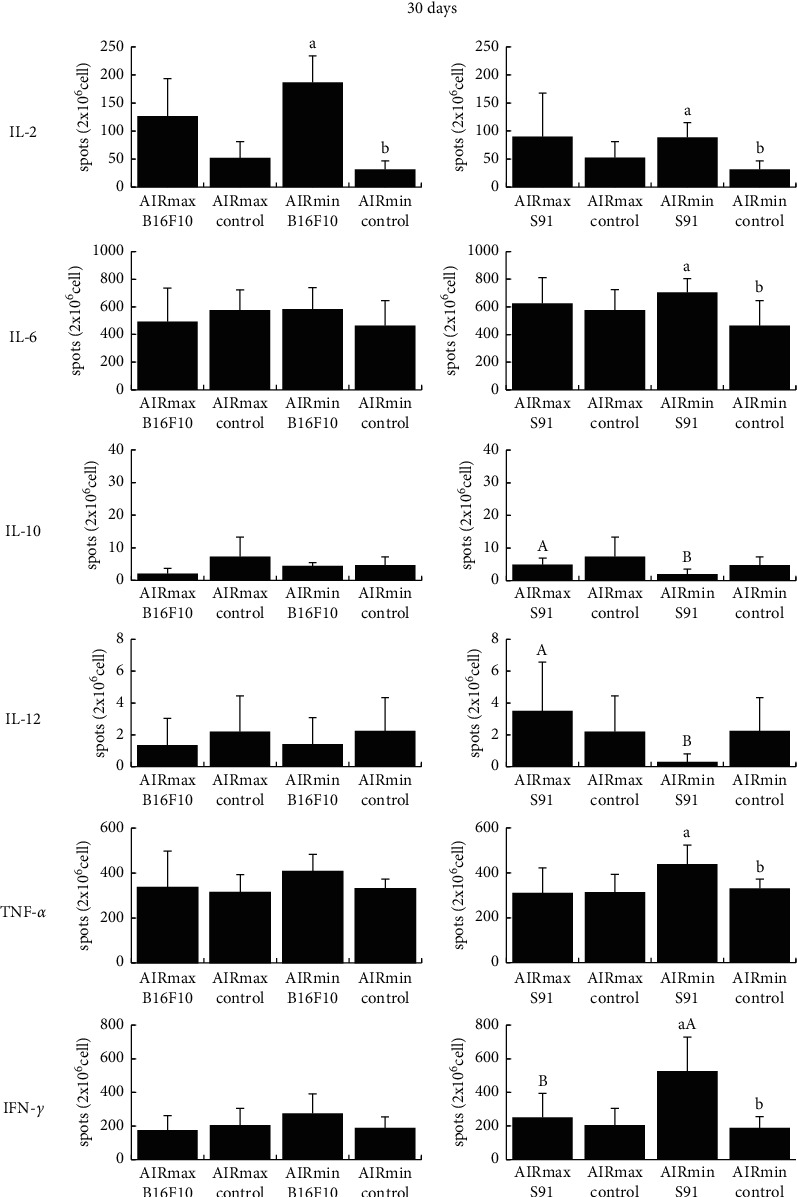
Percentage of the number of cytokine-producing spleen cells. AIR mice lineage were subcutaneously inoculated with melanoma cells (B16F10 or S91), and after 30 days, the numbers of IL-2-, IL-6-, IL-10-, IL-12-, TNF-*α*-, and IFN-*γ*-producing spleen cells were evaluated by the ELISpot assay. Values are expressed as mean spots/2 × 10^6^cel ± SD (*n* = 13). Lowercase letters indicate comparison of cytokine-producing spleen cells percentage within the same AIR mouse line (*a* > *b*, *P* < 0.05); capital letters indicate a comparison between AIRmax and AIRmin (*A* > *B*, *P* < 0.05).

**Figure 3:**
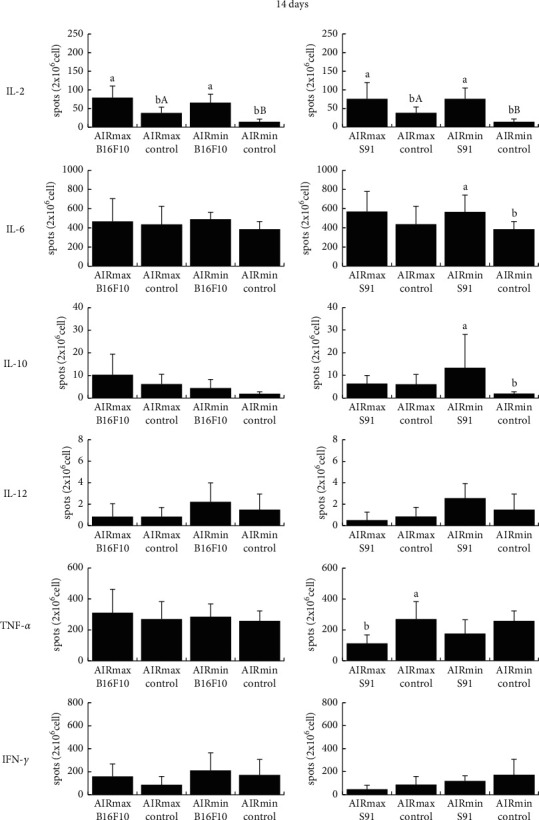
Percentage of the number of cytokine-producing spleen cells. AIR mice lineage were subcutaneously inoculated with melanoma cells (B16F10 or S91), and after 14 days, the numbers of IL-2-, IL-6-, IL-10-, IL-12-, TNF-*α*-, and IFN-*γ*-producing spleen cells were evaluated by the ELISpot assay. Values are expressed as mean spots/2 × 10^6^cel ± SD (*n* = 13). Lowercase letters indicate comparison of cytokine-producing spleen cells percentage within the same AIR mouse line (*a* > *b*, *P* < 0.05); capital letters indicate a comparison between AIRmax and AIRmin (*A* > *B*, *P* < 0.05).

**Figure 4:**
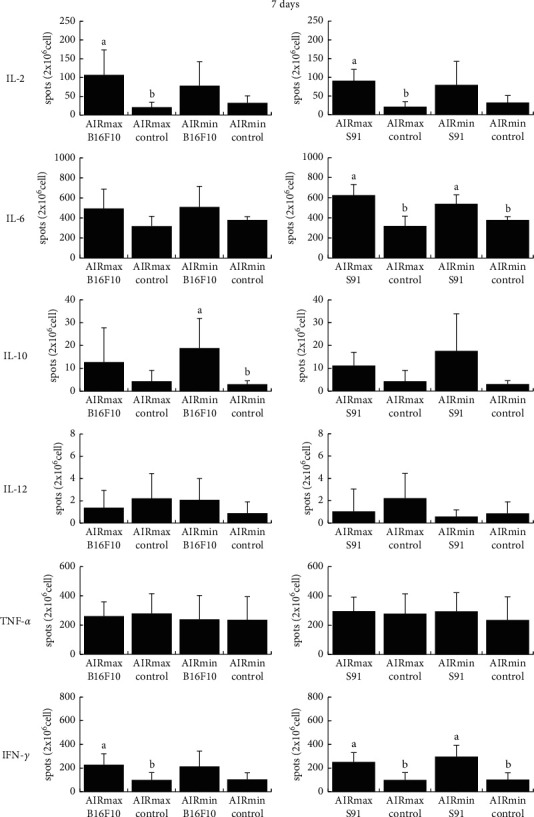
Percentage of the number of cytokine-producing spleen cells. AIR mice lineage were subcutaneously inoculated with melanoma cells (B16F10 or S91), and after 7 days, the numbers of IL-2-, IL-6-, IL-10-, IL-12-, TNF-*α*-, and IFN-*γ*-producing spleen cells were evaluated by the ELISpot assay. Values are expressed as mean spots/2 × 10^6^cel ± SD (*n* = 13). Lowercase letters indicate comparison of cytokine-producing spleen cells percentage within the same AIR mouse line (*a* > *b*, *P* < 0.05); capital letters indicate a comparison between AIRmax and AIRmin (*A* > *BP* < 0.05).

We evaluated the *ex vivo* number of IL-2-, IL-6-, IL-10-, IL-12-, TNF-*α*- and IFN-*γ*-producing spleen cells, since tumor growth can be affected by cytokines. S91 melanoma growth induced a higher number of IL-10-secreting cells in AIRmax mice (4.857 ± 2.015) than in AIRmin mice (1.949 ± 1.565) and considerably more IL-12-producing spleen cells (AIRmax: 3.500 ± 3.082 *versus* AIRmin: 0.278 ± 0.507) at 30 days (Figure 2). In contrast, AIRmax mice showed a lower number of IFN-*γ*-producing spleen cells than that of AIRmin mice (AIRmax: 252.188 ± 141.997 *versus* AIRmin: 526.364 ± 201.966; Figure 2). In the control groups, AIRmax mice showed a higher number of IL-2-producing spleen cells than that of AIRmin mice at 14 days (AIRmax: 38.317 ± 15.267 *versus* AIRmin: 14.279 ± 7.302; Figure 3). No relevant changes were observed in the generation of IL-6- and TNF-*α*-producing cells between AIRmax and AIRmin mice (Figures 2–4).

## 4. Discussion

Phenotypically selected AIR mice have been shown to be a useful murine model for studying the mechanism linking inflammatory processes to multiple outcomes [3–6, 18–21]. Genetic studies have indicated that the contrasting inflammatory response of AIR strains involves at least 11 QTLs (quantitative trait loci), such as *Irm* 1 and *Irm* 2 as well as specific genes/alleles, such as the *Slc11a1* and *Ahr* genes [8–12]. So, we used them to understand the influence of the AIR phenotype on certain lymphocyte subtypes, cytokine-secreting cells, and NK cytotoxicity during the *in vivo* B16F10 and S91 melanoma cell development.

In this study, we used two lineages of transplantable murine melanomas, B16F10 and S91. These cells were chosen based on the H-2 haplotypes, considering that AIRmax mice are predominantly H-2^b^, and AIRmin mice are H-2^d^ [6]. We then chose S91 melanoma (H-2^d^), originally derived from DBA/2 animals, and B16F10 (H-2^b^), derived from C57BL/6 mice [6, 28, 29].

We observed that B16F10 induced a higher number of CD4^+^/CD25^+^ cells in AIRmax mice than in AIRmin mice, whereas an inverse response was observed for the S91 cells. Moreover, S91 promoted a higher number of CD8^+^ cells in AIRmax mice. However, the number of CD49^+^ NK cells and cytotoxic NK activity were similar in both mouse strains.

Previous studies have shown that melanomas are indeed able to modulate the immune system by inducing a specific immune response against tumor-associated antigens (gp100, gp75, MART-1, and tyrosinase) [30, 31] and through the production of growth and immune-regulatory factors, such as fibroblast growth factor, transforming growth factor-*α* and *β* (TGF-*α* and TGF-*β*), IL- 8, and IL-10 [31–33]. Considering the susceptibility of AIRmax mice to B16F10 development and the increased presence of CD4^+^/CD25^+^ cells in the early tumor growth stages, we speculated that CD4^+^/CD25^+^ cells represent the regulatory T cell population, which could suppress the antitumor response, allowing a higher incidence of tumors in AIRmax than in AIRmin mice [34, 35]. Although we have not investigated FOXP-3 expression to confirm the Treg cell phenotype [33] and rule out that these cells might be active T cells, our findings are corroborated by Larocca et al. [36] who observed that AIRmax mice constitutively showed a larger number of CD4^+^/CD25^+^/FOXP-3^+^ Treg cells in their spleens and lymph nodes than that of AIRmin mice.

Studies with the AIR strain have shown that the intense inflammatory response of AIRmax is followed by powerful immunoregulatory mechanisms [36, 37], and the resistance or susceptibility to various diseases depends on the type of immunogenic stimulus [38]. We observed that S91 growth in AIRmax mice, despite showing a reduced number of CD4^+^/CD25^+^ cells and an increased CD8^+^ and IL-12 production compared to that of AIRmin mice, induced a high number of IL-10-producing spleen cells and a lower number of IFN-*γ*-producing cells. Treg cells are essential for maintaining self-tolerance and are crucial for proper functioning of a healthy immune system; however, in the context of cancer, these cells can limit antitumor immune response thereby contributing to an immunosuppressive microenvironment by releasing suppressive cytokines such as IL-10 [39]. Considering the suppressive role of IL-10 and Treg cells [35, 36], these features could explain the inability of AIRmax to maintain cytotoxic activity when challenged with melanoma cells, and consequently, the higher incidence of primary tumors in animals.

Activated CD8^+^ T cells are capable of directly recognizing and killing malignant and infected cells via the exocytosis of cytotoxic granules containing perforin and granzymes as well as production of proinflammatory cytokines such as IFN-*γ* and TNF-*α* [39]. In this context, IL-12 is also an important cytokine for its ability to stimulate the functions of NK cells, macrophages, and T lymphocytes [22]. High CD8^+^ cell, IL-12, and IL-10 levels, and lower IFN-*γ* production in the AIRmax mice were previously observed in different situations [22, 36, 38, 40] and demonstrated a delicate balance between the nature of the stimulus and the host response.

Alternatively, the resistance of AIRmin to melanoma development may reflect a more efficient antitumor immune response. This observation is supported, at least in part, by the reduced number of CD4^+^/CD25^+^ T cells during B16F10 development in accordance with the decrease in IL-10-producing cells and increased number of IFN-*γ*-producing cells. Furthermore, the lower incidence of B16F10 primary tumors in AIRmin mice may have been influenced by major histocompatibility complex incompatibility.

In agreement with our findings, Larocca et al. [36] observed that skin isografts were completely accepted by AIRmax mice. In contrast, skin grafts were rejected by AIRmin mice, and this response was related to increased IFN-*γ* production and a reduced number of Treg cells [36]. These data highlight the findings of our study and demonstrate that the maximal inflammatory response requires intense regulatory mechanisms; otherwise, the AIRmax animals would not survive [36].

Thus, the plasticity and redundancy of the immunologic system hinders experimental studies of the genetic control mechanisms of adaptive and innate immune response components [41]. Therefore, using AIR mice as a murine model to study the immune system relationships and disease development has been shown to be advantageous, as the expression of an extreme inflammatory phenotype while maintaining genome heterogeneity allows a better representation of the natural heterogeneity observed in the human population [16, 42].

## 5. Conclusion

We concluded that AIRmax mice have a higher number of CD4^+^/CD25^+^ T cells and IL-10-producing spleen cells than that of AIRmin mice, and a lower number of IFN-*γ*-producing cells. These results indicate that melanoma cells can induce different responses in AIR mice, suggesting that it may be due to alterations in the regulatory mechanisms in AIRmax mice, such as the action of CD4^+^/CD25^+^ Treg cells and IL-10 production, allowing a higher susceptibility to alografted melanoma development.

## Figures and Tables

**Table 1:** Incidence of primary B16F10 and S91 tumor growth in AIRmax and AIRmin mice.

Mice lineage	Tumoral lineage					
	B16F10 melanoma			S91 melanoma		
	7 days	14 days	30 days	7 days	14 days	30 days
AIRmax	0%^*∗*^^a`^	62.5%^b`^ (5/8)	50% (4/8)	0%^*∗*^	61.53%^#^ (8/13)	58.3%^#^ (7/12)
AIRmin	0%^*∗*^	12.5%^a^ (1/8)	25%^a^ (2/8)	0%^*∗*^	50%^b^ (4/8)	36.3% (4/11)

^*∗*^Tumor diameter <1 mm. A < b (*P* ≤ 0.006); a`< *b*` (*P* = 0.02); ^#^*P* ≤ 0.01 (compared to 7 days after implantation in the same AIR mouse lineage).

**Table 2:** Flow cytometry analysis of lymphocyte populations from the spleen of AIR mice (*n* = 5–8). The results are expressed as a means percentage and standard deviation of 10,000 events acquired from 10^7^ cells/mL suspension.

Lymphocyte subsets	Days	Melanoma					
		B16F10		S91		Control	
		AIRmax	AIRmin	AIRmax	AIRmin	AIRmax	AIRmin
	7	*n* = 7	*n* = 5	*n* = 5	*n* = 5	*n* = 6	*n* = 5
CD3^+^		30.62 ± 5.64	22.83 ± 2.18	24.66 ± 6.23	27.57 ± 5.21	24.84 ± 4.13	28.29 ± 1.77
CD4^+^		20.60 ± 4.39	14.41 ± 0.90	15.76 ± 0.87	17.16 ± 2.91	14.96 ± 1.61	17.57 ± 1.66
CD8^+^		15.13 ± 1.66	12.11 ± 3.98	10.47 ± 1.85	11.40 ± 1.72	16.20 ± 3.20	9.53 ± 4.69
CD4^+^/CD25^+^		3.92 ± 0.58^A^	2.89 ± 0.57^B^	1.48 ± 0.21^B^	2.94 ± 0.66^A^	2.87 ± 0.90	1.99 ± 1.35
CD3^+^/CD49b^+^		2.60 ± 0.40	2.01 ± 0.23	1.21 ± 0.62	1.62 ± 0.23	2.02 ± 0.56	1.77 ± 0.26
CD49b^+^		6.29 ± 0.84	5.90 ± 1.54	3.85 ± 0.85	4.18 ± 1.45	6.01 ± 1.14	6.30 ± 1.90
	14	*n* = 8	*n* = 5	*n* = 8	*n* = 5	*n* = 8	*n* = 5
CD3^+^		32.47 ± 4.65	22.24 ± 2.54	36.86 ± 9.32	27.64 ± 4.56	39.55 ± 5.46^A^	25.89 ± 4.28^B^
CD4^+^		20.25 ± 2.58	15.04 ± 1.40	23.13 ± 4.96	18.23 ± 3.58	23.58 ± 2.92	16.76 ± 4.48
CD8^+^		12.46 ± 3.34	6.72 ± 1.17	14.38 ± 4.24^A^	8.07 ± 0.90^B^	15.20 ± 2.22^A^	9.04 ± 2.24^B^
CD4^+^/CD25^+^		2.64 ± 1.14^A^	0.87 ± 0.38^B^	2.64 ± 0.91	2.21 ± 1.29	2.90 ± 1.00	1.38 ± 0.86
CD3^+^/CD49b^+^		3.67 ± 0.99	3.23 ± 0.94	4.01 ± 0.87	3.22 ± 0.98	3.90 ± 1.22	3.94 ± 0.91
CD49b^+^		3.31 ± 0.51	4.61 ± 1.19	4.12 ± 0.85	4.58 ± 1.71	3.51 ± 1.01	3.98 ± 1.29
	30	*n* = 5	*n* = 5	*n* = 5	*n* = 5	*n* = 5	*n* = 5
CD3^+^		30.69 ± 4.71^a^	24.12 ± 3.20	18.62 ± 4.95^b^	22.86 ± 2.45	32.94 ± 2.45^a^	23.66 ± 2.43
CD4^+^		19.65 ± 1.56^a^	17.29 ± 4.47	12.00 ± 2.97^b^	15.18 ± 1.27	22.07 ± 4.47^a^	16.82 ± 1.70
CD8^+^		11.91 ± 2.97^a^	8.84 ± 1.66	6.79 ± 1.96^b^	7.93 ± 2.00	10.62 ± 2.29	7.06 ± 2.22
CD4^+^/CD25^+^		2.60 ± 0.65	1.92 ± 0.75	1.42 ± 0.48	1.49 ± 0.49	2.51 ± 0.76	1.24 ± 0.69
CD3^+^/CD49b^+^		2.11 ± 0.83	1.33 ± 0.06	1.39 ± 0.88	1.26 ± 0.21	1.34 ± 0.19	1.30 ± 0.47
CD49b^+^		4.86 ± 0.65	3.79 ± 0.73	4.25 ± 0.86	4.28 ± 0.40	3.91 ± 0.26	3.92 ± 0.72

Lowercase letters indicate the comparison of lymphocyte subsets within the same AIR mouse line (a > b, *P* < 0.05). Capital letters indicate a comparison of lymphocyte subsets between AIRmax and AIRmin (A > *B*, *P* < 0.05).

## Data Availability

The raw data are available from the corresponding author upon reasonable request.
